# A Replication Crisis in Mathematics?

**DOI:** 10.1007/s00283-020-10037-7

**Published:** 2021-03-05

**Authors:** Anthony Bordg

**Affiliations:** University of Cambridge, Cambridge, England

According to the mathematician Vladimir Voevodsky, mathematics is on the verge of a crisis:

Seeing how mathematics was developing as a science, I understood that the time is approaching when the proof of yet another conjecture will change little. I understood that mathematics is facing a crisis ... [it] has to do with the complication of pure mathematics which leads, again sooner or later, to articles becoming too difficult for detailed proofreading and to the start of unnoticed errors accumulating. And since mathematics is a very deep science, in the sense that the results of a single article usually depend on results of very many previous articles, this accumulation of errors is very dangerous.^1^

Since a crisis is an unexpected event coming as a surprise to communities and organizations, insiders often being slower than outsiders to diagnose its onset due to denial, one may want to pause in order to ponder Voevodsky’s claims. The interview from which this quotation is extracted took place in 2012, namely 10 years after Voevodsky was awarded the Fields Medal for remarkable mathematical developments culminating in his celebrated proof of the Milnor conjecture [4].

The word “crisis” derives from the Ancient Greek “krísis,” meaning an unstable situation or a turning point, and from the verb “krínein,” meaning to decide. What could be the unstable or unsustainable situation faced by mathematicians? What would be the turning point? What should mathematicians do if they are on the verge of a crisis?

We will focus in this article on this alleged crisis, namely the increasing difficulty in checking the correctness of mathematical arguments. As pointed out by Voevodsky, this could be a real danger, mathematics being a cumulative endeavor, a cathedral of proofs. Certainly, if the outputs of curiosity-driven mathematical research may not be immediately useful, they should be at least correct.

## The Fall of the Peer Review System

In this section I propose to investigate the symptoms of the alleged crisis developed by mathematics.

We will begin with Voevodsky himself, and in his own words, learn what led him to the conclusions reported above. In a letter [11] subtitled “A Personal Mission to Develop Computer Proof Verification to Avoid Mathematical Mistakes,” Voevodsky tells us how he witnessed mistakes taking years to uncover in his own mathematical work through his journey to develop the kind of higher-dimensional mathematics envisioned first by Alexander Grothendieck. He reports similar issues affecting the work of some of his brilliant colleagues. Reflecting on these issues, Voevodsky concludes that

A technical argument by a trusted author that is hard to check and looks similar to arguments known to be correct is hardly ever checked in detail.

One reason for this unfortunate state of affairs is mathematical research currently relying “on a complex system of mutual trust based on reputations.” In other words, mathematicians rely partly on social processes, such as reputation and academic credentials, for verification, with the risk of incorrect results being unnoticed and then used in further developments. We should emphasize that these not uncommon mistakes happen not only in preprints but also in the published literature, this “mutual trust based on reputations” having an influence on the peer review process. Worse, faulty published papers are not always retracted. The fact that today, preprints are widely available in the online repository arXiv, sometimes before the peer review and publication processes, does not help, and it makes Voevodsky’s warning even more important in today’s fast-paced research.

The solution advocated by Voevodsky consists in using computers to formally check mathematical proofs. Among mathematicians, Voevodsky credits Thomas Hales and Carlos Simpson with trying to advance the field of computer verification in mathematics when he began to explore the possibility of computer proof verification, which was at that time “almost a forbidden subject among mathematicians.”

Thomas Hales’s foray into the field of formal verification of mathematical proofs, despite his being a mathematician working in unrelated fields such as representation theory and discrete geometry, is telling. In “Formal Proofs” [5], Hales reflects on the unfolding of the peer review process after he submitted to the *Annals of Mathematics* his proof of the Kepler conjecture, a conjecture about optimal sphere packing asserting that “no packing of congruent balls in three-dimensional Euclidean space can have density greater than the density of the face-centered cubic packing.” In layman’s terms, the conjecture asserts that the best way to stack oranges on a market shelf is a pyramidal arrangement, something that any greengrocer would vouch for. However, the conjecture, first stated by Johannes Kepler in 1611, remained open for roughly 400 years. Having worked hard for a very long time and run a seminar at Eötvös Loránd University over a three-year period, the referees were ultimately unable to certify the correctness of the proof. In addition to a 300-page text, the proof consisted of about 180,000 lines of custom, uncertified computer code [6] to exhaust different configurations of spheres. According to Hales, the code was never scrutinized by the referees; they only assessed that the methods involved were a priori strong enough for them to be confident in the validity of the proof. While the truth of the Kepler conjecture may have been established by Ferguson and Hales in 1998, their proof was not published until 2006. In the meantime, to circumvent the referees’ difficulties in checking the proof and to clear away any remaining doubts, Hales embarked in 2003 on the Flyspeck project to give a certified proof of the Kepler conjecture using a proof assistant, a piece of software for writing formal proofs, every step of which is checked by the software and completely justified by the elementary rules of the underlying logical system. The formal proof was completed in 2014, and we learned that “hundreds of small errors in the proof of the Kepler conjecture were corrected during formalization” [7]. Interestingly, the formal proof follows the same general outline of the original proof, but various amendments were made. Hales wrote:

Because the original proof was not used for the formalization, we cannot assert that the original proof has been formally verified to be error free. Similarly, we cannot assert that the computer code for the original proof is free of bugs [7].

In other words, proofs that are not beyond reasonable doubt, either due to the sheer scale of the reviewing task or due to gaps that cannot be easily filled, will remain in Purgatory even when the methods involved in the attempt are strong enough to lead later to a mechanized proof written with a proof assistant. After all, the authors of those methods remain human, hence fallible, even if their methods are not. Thus, only formal proofs seem to be destined for Paradise, while some proofs may remain in Purgatory before sometimes being condemned to Hell when mistakes are uncovered or gaps are deemed impossible to fill. Thus, contrasting with the platonism often assumed by mathematicians with respect to mathematical objects, mathematical proofs seem to have a temporal component displayed in a dramatic remake of the *Divine Comedy*. With the case of the so-called *abc* conjecture, we will shortly see below that mathematical proofs can have a geographical component as well, with something being a proof in Japan and not being a proof elsewhere. This spatiotemporal status of mathematical proofs could lead a playful theologian to argue in favor of a “dispensability” argument, concluding that proofs are dispensable, God directly intuiting the truth of mathematical statements, and Erdős’s Book^2^ would be nowhere to be found. After all, if God exists, he is also a physicist, not only a mathematician.^3^ However, the Book could well be written in the future on a computer.

In August 2012, the noted Japanese mathematician Shinichi Mochizuki, professor at Kyoto University, released online a series of four papers developing a theory he called “inter-universal Teichmüller theory” (IUT). According to the abstract of the fourth paper [8], this work provides among other results a proof of the *abc* conjecture. This is a central conjecture in number theory, also known as the Oesterlé–Masser conjecture, first proposed in 1985 with numerous consequences; in particular, it implies Fermat’s last theorem. Despite several international conferences organized since then, as of October 2020, the mathematical community has still not reached agreement on the correctness of Mochizuki’s proof. Mochizuki’s series of four papers amounts to 500 pages of mathematics. Moreover, his papers build on highly nontrivial prerequisites in a field called arithmetic anabelian geometry. According to Ivan Fesenko, the total volume of relevant papers in anabelian geometry used in Mochizuki’s work amounts to 1,500 pages [3].

While some colleagues and some close collaborators of Mochizuki’s are convinced that his proof is correct, two distinguished German mathematicians, Peter Scholze and Jakob Stix, believe they have pinpointed a flaw [9]. So, we end up now with the absurd situation that *abc* is a theorem in Japan while still an open conjecture in Germany. The mathematical community has still not properly settled the case after all these years despite Fesenko noting that

The total amount of time dedicated to the verification process of IUT by mathematicians already exceeds 30 researcher-years. This definitely looks to be the largest time ever spent in the history of mathematics on the verification of a mathematical work prior to its publication [3].

Nowadays, the status of a mathematical proof becomes unstable and subject to revision. The mental states of mathematicians become unstable too, since they can doubt their own work, worrying about their proofs being too complicated or about the best way to convince others that their proofs are correct. Given the sheer scale of some modern proofs, the peer review system in its present form seems unsustainable.

## The Rise of Silicon Reviewers?

It is perhaps clear now why it may be legitimate to claim, as Voevodsky did, that mathematics is on the verge of a crisis. It is also clear that the turning point may have already passed. It happened, perhaps, when the peer review system broke down, as it did in the process of verifying Hales’s proof of the Kepler conjecture. In that case, a working mathematician, Thomas Hales, decided to use a proof assistant to mechanically check his proof. This crisis, if it is indeed one, is not so much a crisis of mathematics itself as a crisis of its peer review system, but it could force mathematics to reinvent itself. Also, it could be the prelude to a more serious crisis when artificial intelligence will have caught up with human mathematics, should that ever happen.

At that stage, some serious discussions will be necessary. Can we really trust a proof assistant to check our mathematical proofs? After all, it is well known that every computer program contains bugs; hence it may not be enough to prove the soundness of the underlying logic of the system: its implementation matters. First, note that the kernel of such a proof assistant, namely the core part that implements its rules of inference, is small, perhaps a few thousand lines of code, sometimes only a few hundred for less-expressive logics, far smaller that the operating system you use on your computer. So one has confined the risk of errors in this small amount of code, a notable advance. Second, formal methods are used not only for mathematical proofs, but also for software and hardware verification, and it is possible to use another proof assistant with a more expressive logic to check the kernel of a first proof assistant, bypassing Gödel’s second incompleteness theorem. In this way, one can reduce the risk of errors in mathematical proofs by several orders of magnitude compared with the verification process of even the most prestigious mathematics journals, which rely on human and social processes. Thus, proof assistants could allow mathematicians to be more faithful to their ideal of rigor. However, it is true that the plan outlined above implies a kind of regression: I am afraid that absolute certainty is an ether too rare for humans to breathe.

We should emphasize that human peer review will remain necessary. Indeed, the peer review system has two functions, not only to ensure the correctness of mathematical proofs, but also to evaluate the mathematical value of new results, and therefore, humans will remain key players in deciding what results are worthy of publication. By using proof assistants to formally check the correctness of mathematical proofs, one alleviates the work of human reviewers, which allows them to focus on the second function of the peer review process.

The two functions of the peer review process alluded to above correspond to two functions of mathematical proofs in written communications. On the one hand, they communicate ideas, intuitions, and ways of thinking about mathematical objects, as eloquently argued by William Thurston [10]. On the other hand, a mathematical proof can be thought of as a set of instructions that allow readers to replicate the proof, hence convincing themselves of its correctness. This act of replication can be indirectly judged against an ideal personal proof that would unfold from theorems that are firmly held to be true by the reader. In other words, the replication of scientific results is as relevant in mathematics as it is in the natural sciences.

If mistakes in proofs prevent this act of replication, serious gaps in them damage it. Given that mathematicians vary to a large degree in how comfortable they are omitting details, gaps impair their ability to replicate proofs for themselves. The formal verification of proofs can be seen as a way to enforce a gap reduction to make sure that no one is left behind. One could say that the formal verification of a mathematical proof performs the function of replication once and for all, and it allows the reader to focus on the ideas and intuitions behind the proof. If one does not want these two functions to be split, then formal libraries of mathematics will have to be much more legible than they presently are.

I would like to disagree with Thurston’s assertion, “We have good human processes for checking mathematical validity” [10]. Thurston is rather lucid on a number of points, noting that “The standard of correctness and completeness necessary to get a computer program to work at all is a couple of orders of magnitude higher than the mathematical community’s standard of valid proofs.” But from his next sentence, “Nonetheless, large computer programs, even when they have been very carefully written and very carefully tested, always seem to have bugs,” it is clear that Thurston is talking about uncertified computer programs. With computer programs certified with formal methods, this standard of valid proof is again at least a couple of orders of magnitude higher. As noted by Thurston, this new standard has a cost:

Mathematics as we practice it is much more formally complete and precise than other sciences, but it is much less formally complete and precise for its content than computer programs. The difference has to do not just with the amount of effort: the kind of effort is qualitatively different. In large computer programs, a tremendous proportion of effort must be spent on myriad compatibility issues: making sure that all definitions are consistent, developing “good” data structures that have useful but not cumbersome generality, deciding on the “right” generality for functions, etc. The proportion of energy spent on the working part of a large program, as distinguished from the bookkeeping part, is surprisingly small. Because of compatibility issues that almost inevitably escalate out of hand because the “right” definitions change as generality and functionality are added, computer programs usually need to be rewritten frequently, often from scratch.

A very similar kind of effort would have to go into mathematics to make it formally correct and complete .... It is also quite hard to come up with good technical choices for formal definitions that will be valid in the variety of ways that mathematicians want to use them and that will anticipate future extensions of mathematics.

It is certainly true, as noticed by Thurston, that formal proofs force mathematicians to process very low-level details and keep track of the bookkeeping part with tremendous effort. However, the creation and development of formal languages such as dependent type theories, which made possible recent advances in the foundations of mathematics like Voevodsky’s Univalent Foundations [2], could ease the formal verification of mathematical proofs in the future.

In sum, mathematicians have not massively adopted the technology offered by proof assistants. Indeed, much progress remains to be made in order to make them more user-friendly, although it is indisputable that formal proofs set a new benchmark for the mathematician’s ideal of rigor.

If mathematics is on the verge of a crisis, it is not a crisis of mathematics itself. Mathematics is healthy and robust. It is rather a crisis of its peer review system. However, this crisis could be very problematic should the situation worsen, peer review being instrumental in the workings of the mathematics community. From this perspective, it is worthwhile to make further progress in the formalization of mathematics using proof assistants in order to make the peer review process smoother.

## Footnotes

1 Interview with Vladimir Voevodsky by Roma Mihailov, published in Russian on the LiveJournal account baaltii1 (July 1, 2012), translation courtesy of Innokentij Zotov: http://baaltii1.livejournal.com/198675.html.

2 See the preface of the book *Proofs from THE BOOK* [1].

3 Physicists are notorious for not practicing the art of mathematical proof.
